# Biologic Mesh Reconstruction of the Pelvic Floor after Extralevator Abdominoperineal Excision: A Systematic Review

**DOI:** 10.3389/fsurg.2016.00009

**Published:** 2016-02-16

**Authors:** Nasra N. Alam, Sunil K. Narang, Ferdinand Köckerling, Ian R. Daniels, Neil J. Smart

**Affiliations:** ^1^Exeter Surgical Health Services Research Unit (HeSRU), Royal Devon and Exeter Hospital, Exeter, Devon, UK; ^2^Department of Surgery, Center for Minimally Invasive Surgery, Academic Teaching Hospital of Charité Medical School, Vivantes Hospital, Berlin, Germany

**Keywords:** ELAPE, extralevator abdominoperineal excision of rectum, extralevator abdominoperineal resection, pelvic floor reconstruction, biological mesh

## Abstract

**Introduction:**

The aim of this review is to provide an overview of the evidence for the use of biologic mesh in the reconstruction of the pelvic floor after extralevator abdominoperineal excision of the rectum (ELAPE).

**Methods:**

A systematic search of PubMed was conducted using the search terms: “ELAPE,” “extralevator abdominoperineal excision of rectum,” or “extralevator abdominoperineal resection.” The search yielded 17 studies.

**Results:**

Biologic mesh was used in perineal reconstruction in 463 cases. There were 41 perineal hernias reported but rates were not consistently reported in all studies. The most common complications were perineal wound infection (*n* = 93), perineal sinus and fistulae (*n* = 26), and perineal haematoma or seroma (*n* = 11). There were very few comparative studies, with only one randomized control trial (RCT) identified that compared patients undergoing ELAPE with perineal reconstruction using a biological mesh, with patients undergoing a conventional abdominoperineal excision of the rectum with no mesh. There was no significant difference in perineal hernia rates or perineal wound infections between the groups. Other comparative studies comparing the use of biologic mesh with techniques, such as the use of myocutaneous flaps, were of low quality.

**Conclusion:**

Biologic mesh-assisted perineal reconstruction is a promising technique to improve wound healing and has comparable complications rates to other techniques. However, there is not enough evidence to support its use in all patients who have undergone ELAPE. Results from high-quality prospective RCTs and national/international collaborative audits are required.

## Introduction

Abdominoperineal excision of the rectum (APER) is used as a treatment modality in patients with rectal cancer where an anterior resection (AR) and an anastomosis cannot be performed (1). Extralevator abdominoperineal excision (ELAPE) involves the en bloc excision of the levator muscles and the rectum, in order to reduce the risk of tumor involvement in the circumferential resection margins (CRMs) and reduce the risk of tumor perforation intraoperatively. This method has been demonstrated as leading to a wider surgical margin and therefore fewer positive CRMs (2–5). Initially, the terminology used was “*cylindrical APER*” but with refinement and the use of MRI to highlight the area of risk of a positive CRM, the term ELAPE is more appropriate (4). The nomenclature surrounding the technique has been the source of much debate and confusion, with some authors noting that ELAPE is no different from the original description in English by Miles (6). Furthermore, what exactly constitutes “standard” surgery that allows differentiation of ELAPE has come under scrutiny (7).

Volumetric analysis has confirmed that ELAPE does remove more tissue (3), and the wider excision can, however, increase morbidity and wound complications and will require some form of perineal reconstruction (4). Perineal wound problems are reported in up to 57% of patients undergoing APER (8), although the precise rates following ELAPE are not yet known. Given that ELAPE produces a larger defect in the pelvic floor, leaving only the ischiorectal fat and skin to close the perineal wound; it is presumed that the perineal complication rate is higher. Furthermore, the changes in the proportion of patients having neoadjuvant (chemo)radiotherapy over the time course of ELAPE implementation are incompletely reported in individual studies and in national registries. If the wound fails to heal *via* primary intention, secondary wound healing can result in prolonged hospital stay that requires intensive wound care.

**Figure 1:**
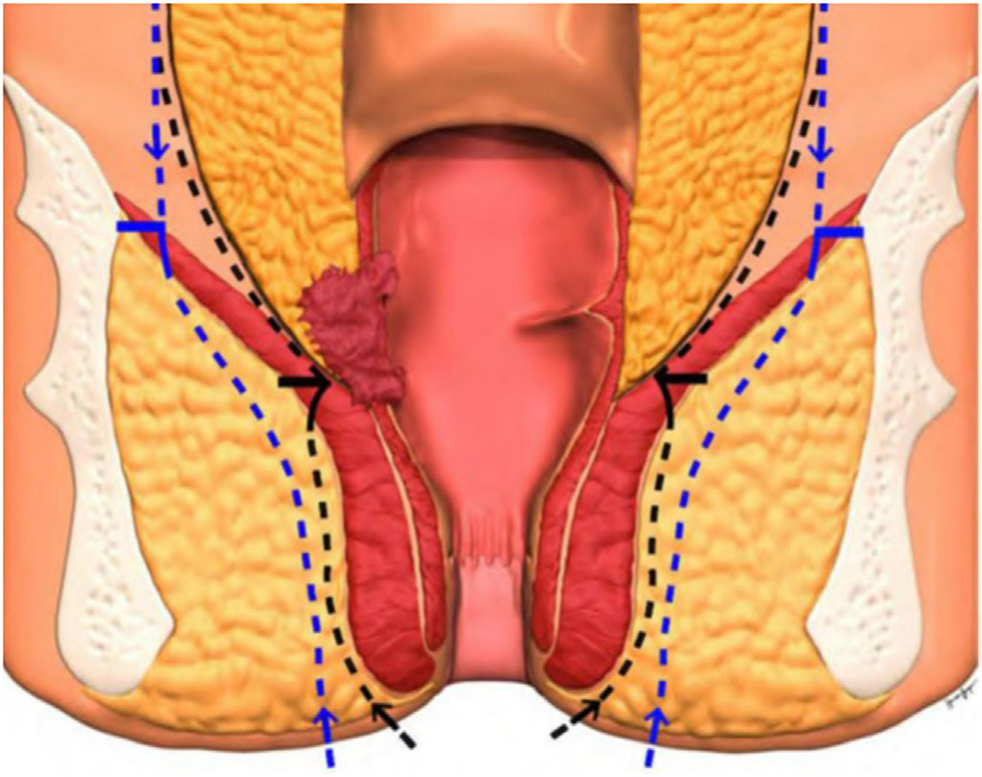
**ELAPE technique (13)**. Black line indicates dissection line of standard APE and blue line ELAPE. Horizontal line indicates meeting point of abdominal and perineal dissection.

Various alternative techniques have been described to reconstruct the pelvic floor following ELAPE with the aim to reduce perineal wound complications and hernias. The optimal method of perineal reconstruction remains a matter of debate. Myocutaneous flaps, such as those derived from gluteus maximus (2, 4, 9), rectus abdominis, and latissimus dorsi muscles (4, 10), have been used but are associated with donor-site morbidity, flap necrosis, prolonged operative time, additional resources, and increased cost. Biologic mesh has recently been introduced as an alternative form of reconstruction in order to improve perineal wound healing and reduce perineal hernia rates (11). The mesh is usually placed as an inlay or bridge across the defect in the pelvic floor in close relation to the bony structures and sutured in 1-cm intervals to the origin of the levator muscles laterally (12). [Figure 1 (13)] The mechanism by which the use of a bridging prosthesis reduces perineal wound problems is not clear. It has been suggested that biological mesh allows native cellular ingrowth and promotes tissue remodeling, which in turn reduces perineal wound problems (14, 15). Alternatively, the biologic mesh may act as a physical barrier, supporting the pelvic contents (omentum, small bowel, and uterus) and minimizing the pressure on the skin and ischiorectal fat as they heal.

Alternative methods for removing the pressure of small bowel that prolapses into the pelvis, directly on the perineum include the following:

(1) Omental pedicle flaps (16–18),
(2) Mobilization of the cecum (9),
(3) Retroversion of the uterus in female (19).

All of the above techniques are designed to close off the dead space in the pelvis, resulting from the removal of the rectum and to keep the small bowel out of the pelvis. Of these methods, the most widely established is the omental pedicle. However, these techniques largely related to an era of open surgery, and they have mostly been abandoned with the move to laparoscopic and other minimally invasive techniques and are not representative of contemporary practice. Omental pedicles are associated with perineal wound complication rates of 14–18% and decreased wound dehiscence in comparison to primary closure (16, 18) whereas others show no advantage to this technique (20). Mobilization of the cecum is uncommon and evidence is limited to case reports (9). Retroversion of the uterus involves retroverting the uterus and securing it to the bony pelvis at a level where it obliterates the pelvis, with the use of non-absorbable suture material (19). This can be achieved *via* the abdominal or perineal wound, although it has been associated with dyspareunia and positional menstruation (19).

The aim of this review is to provide an overview of the evidence for the use of biologic mesh in the reconstruction of the pelvic floor after extralevator abdominoperineal excision.

## Methods

A systematic search of PubMed was conducted using the search terms: “ELAPE,” “extralevator abdominoperineal excision of rectum,” or “extralevator abdominoperineal resection” in order to identify studies evaluating the use of biologic mesh for reconstruction of the pelvic floor. Titles, abstracts, and full texts were analyzed for studies reporting on the use of biologic mesh for reconstruction of the pelvic floor. Inclusion criteria were studies that used biologic mesh for perineal reconstruction. Studies were excluded if only synthetic mesh was used or if there was no mention of a mesh. Furthermore, studies on patients under the age of 18 were excluded as well as non-English language studies, technical tips, conference abstracts, or duplicates series from the same research group. Overall, the search yielded 17 studies for analysis after the exclusion of review articles. The study characteristics are presented (Table 1).

**Table 1:** **Reconstruction of the pelvic floor after ELAPE**.

Reference	Study design	No. of pts	Age	Sex (M:F)	Patient characteristics	Material used	Intervention	Follow-up (months)	Complications	LoE
Christensen et al. (21)	Case series	57	FLAP: 67.8 (32.7–86.2)	11:22	52 primary rectal cancer 5 local recurrence 48 patients (84%) received neoadjuvant CRT	Gluteal flaps: 33	ELAPE for low rectal cancer	Median follow-up: gluteal flap: 3.2 years (1.7– 4.3)	Gluteal flap vs. biologic Perineal hernia: 7 vs. 0, *P* < 0.01 Infectious complications: 2 (17%) vs. 4 (6%), *P* < 0.26 1 patient per group with a persistent perineal sinus	4
			MESH: 69.7 (48.7–84.5)	10:14		Permacol: 24		Biologic mesh: 1.7 (0.4 –2.2) years		
Dalton et al. (22)	Case series	31	Mean 66.8 ± SD 11.4 years	8:23	Neoadjuvant CRT: 14	VRAM flap: 1 Permacol: 30	Open ELAPE	Median: 20 (0–45)	Breakdown of perineal wound: 6 Skin paddle necrosis of a VRAM flap: 1 Perineal wound hematoma: 1 Minor wound discharge: 9	4
Han et al. (23)	Case series	12	68 (49–80)	7:5	Ultra low rectal cancer. Neoadjuvant CRT: 3	HADM	Cylindrical APR-open	Median: 8 (2–16)	Asymptomatic seroma: 1 Perineal wound infection: 1	4
Han et al. (14)	Open label RCT	67	63 median (44–81)	20:15	Neoadjuvant therapy: 10	HADM	ELAPE: 35	Median: 29 (12–48)	Bowel perforation: 2 Perineal wound infection: 4 Perineal seroma: 4 Peristomal hernia: 16 Abdominal wound infection: 2 Perineal herniation: 5	2
			68 (32–84)	21:11	Neoadjuvant therapy: 9	None	APER: 32	Median: 22 (14–46)	Bowel perforation: 5 Perineal wound infection: 6 Peristomal hernia: 13 Abdominal wound infection: 3 Perineal herniation: 4	
Han et al. (24)	Multicenter prospective cohort study (case series)	109 (102)	61 years (27–78)	60:42		HADM	Biological mesh: 83 (81.4%) Primary closure: 19 (18.6%)	44 median (18–68)	Biological mesh Perineal wound complications: 15 Infection: 5 Seroma: 5 Hernia: 4 Abdominal wound infection: 3 Primary closure Perineal wound complications: 9 Infection: 3 Seroma: 1 Hernia: 2 Wound dehiscence: 3 Chronic sinus: 1 Abdominal wound infection: 2	4
Jensen et al. (25)	Case series	53 – 31 agreed to long-term f/u	69 (33–83) median	33:20	Neoadjuvant CRT: 23	Permacol	6 planned open 47 laparoscopic of which 7 converted to open	Median: 36 (1–67)	Perineal hernia: 3 Fistuale: 11 Perineal abscess: 4 Superficial wound infections: 4 Removal of mesh: 1 Implantation of new mesh: 1	4
Kipling et al. (26)	Case series	28	70 (52–81 years) median	20:8	Neoadjuvant therapy None: 9 (32%) Short course: 2 (7%) Long course: 17 (61%)	Permacol	Lap ELAPE, 5 conversions	Median 38 (23–66)	Bowel perforation: 1 Persistent perineal sinus at 6 months: 1 Delayed healing of the perineal wound: 1	4
Peacock et al. (15)	Case series (comparative)	15	68 median (48–74)	4:1	Long-course CT/RT: 4 Long-course RT: 1	VRAM: 5	Cylindrical APER	Median: 29 (23–35)	Perineal wound infection (wound dehiscence): 1 Flap necrosis: 1 Wound hematoma: 1	4
			57 median (47–68)	9:1	Long-course CT/RT: 6 Long-course RT: 2 (not suitable for CT): 2	Surgisis: 10		13 (3–27)	Perineal sinus: 1 Superficial perineal wound infection: 2 Abscess/collection: 3	
Peacock et al. (27)	Case series	34	Median 62 years (40–77)	27:7	Long-course CRT: 26 Long-course RT (not suitable for CT): 2 Not required/declined: 6	Surgisis:	Cylindrical APER	Median: 21 (1–54)	Perineal sinus: 5 Superficial perineal wound infection: 3 Abscess/collection: 3 Parastomal hernia: 1	4
Vaughan-Shaw et al. (28)	Case series (case–control)	16	71 (49–88)	7:9	Short-course RT: 7 Long-course CRT: 9	9 Permacol/Surgisis (omentoplasty: 7)	Laparoscopic ELAPE: 14 (1 conversion)		Return to theater (<30 days): 2 Perineal wound complications: 2	4
		10	72 (52–87)	5:5	Short-course RT: 7 Long-course CRT: 2		Open: 2 Lap APER: 10 Open APER: 10		Perineal wound complications: 5 Perineal hernia: 2 Infection: 1
		10	72.5 (46–89)	8:2	Short-course RT: 2 Long-course CRT: 5				Return to theater (<30 days): 1 In-hospital mortality: 1 Perineal wound complications: 2
Wille-Jørgensen et al. (29)	Case series	11	63 median (51–77)	7:4	Neoadjuvant CRT: 6	Permacol	Laparoscopic APER: 9 (2 conversions) Open APER: 2	Median: 12 (3–18)	Mesh removal 2nd to infection: 1 Rectal perforation: 1 Long-lasting perineal pain: 6 Fistula: 1	4
Chi et al. (30)	Case series	6	Mean: 69	4:2	Neoadjuvant CRT 4	HADM		Mean: 5 (2–19)	Surgical site infection: 2	4
Palmer et al. (31)	Case series	193	66 median (28–87)	81:112	Neoadjuvant CRT: 91 RT alone: 92 Locally advanced tumor on MRI (T4)-126 (65%)	Perineal closure Gluteal flap: 99 (51) Biological mesh: 66 (34) Closure directly: 28 (15)	Pelvic exenteration: 25, extended resection with parts of other organs: 56 ELAPE alone: 112	Median 31 (0–156)	Intra-operative perforation: 19 30-day postoperative mortality: 6	4
West et al. (4)	Retrospective case series (multicenter)	176	66 (58–73) Median	116:54 6-unknown	Neoadjuvant RT Yes: 135 No: 35 Unknown: 11 Neoadjuvant CT Given: 84 Not given: 81 Unknown: 11	Gluteus maximus: 60 Rectus abdominis: 12 Latissimus dorsi: 1 Permacol: 11	ELAPE: 176 Open surgery: 122 Laparoscopic surgery: 19 Unknown: 35	*NS*	Wound complications Yes: 57 Infection/breakdown/sinus: 41 Perineal hernia: 5 Other: 11	4
		124	68 (57–75) median	87:37	Neoadjuvant RT Yes: 90 No: 24 Unknown: 10 Neoadjuvant CT Given: 48 Not given: 66 Unknown: 10		APER: 124 Open surgery: 56 Laparoscopic surgery: 4 Unknown: 64	*NS*	Wound complications Yes: 11 Infection/breakdown/sinus: 7 Perineal hernia: 1 Other: 3 Unknown: 26
Harries et al. (32)	Prospective case series	48	Median: 63 (40–86)	36:12	Neoadjuvant treatment: 43	Permacol	ELAPE Lap: 28 Conversion: 7 Open: 23	Median: 27 (1–85)	Specimen perforation: 3 (6.4%) Unhealed at 6 months: 4 (8.3%) Perineal sinus: 7 Abdominal wound dehiscence: 1 Ureteric injury: 1 Radiological drainage of pelvic collections: 2 Perineal wound infections: 9	4
Kavanagh et al. (33)	Case report	1	72	0:1	Long-course CRT	Permacol	Lap ELAPE	12	NS	4
Sayers et al. (34)	Case series	54	Median: 69.5 (31–90)	40:14	Neoadjuvant CRT: 52	Primary closure: 46 Bio: 2 FLAP: 6 (VRAM: 5 Gracilis: 1)	Lap ELAPE: 20 Open: 34	Median: 38 (9–61)	Perineal complications: 24 Perineal hernia: 14 Perineal hematoma: 1 Infected myocutaneous flap: 1 Total dehiscence of the perineum: 1	4

*APER, abdominoperineal excision of the rectum; CRT, chemoradiotherapy; CT, chemotherapy; ELAPE, extralevator abdominoperineal excision; HADM, human acellular dermal matrix; LoE, level of evidence; RCT, randomized controlled trial; RT, radiotherapy; VRAM, vertical rectus abdominis muscle*.

## Results

There were 15 case series, one randomized control trial (RCT), and one case report identified. A biologic mesh was used in perineal reconstruction in 463 cases. The different types of biologic mesh used were cross-linked porcine dermal collagen (Permacol™) in 206 cases, 44 using porcine intestinal submucosa (Surgisis©), 136 using human acellular dermal matrix, and 9 using a combination of Permacol™ and Surgisis©. Two studies did not specify the type of biologic mesh used.

### Perineal Hernia

There were 41 perineal hernias reported, but rates were not consistently reported in all studies. In those studies that did report perineal hernia rates, it was difficult to delineate whether hernias occurred in patients that had perineal reconstruction using a biological or synthetic mesh or a myocutaneous flap.

### Perineal Wound Infection/Healing Problems

Perineal wound infection was reported explicitly in 93 cases, whereas the overall rate of perineal problems was much higher. Perineal sinus and fistulae were reported in 26 cases, with a further 11 cases of perineal hematoma or seroma. Some studies have described “perineal wound complications” but not specified whether they were related to infection, dehiscence, hernia, or pain (Table 1).

The most common complications were perineal wound infection and perineal sinus. However, there are no standardized measures for reporting perineal outcomes of any type following ELAPE. Definitions of wound infection, wound healing problems, perineal herniation, pain measurement, and functional status assessment are inconsistent between studies, thus limiting comparisons.

There are very few studies comparing the use of biologic mesh for perineal reconstruction for ELAPE. Two case series compared biologic mesh with myocutaneous flaps and one series compared laparoscopic ELAPE with laparoscopic and open APER. However, they are all of low-level evidence (level 4). Only one RCT was identified that compared patients undergoing ELAPE with perineal reconstruction using a biological mesh, with patients undergoing a conventional APER with no mesh. There was no significant difference in perineal hernia rates or perineal wound infections between the two groups.

## Discussion/Summary

The use of ELAPE over conventional APER is becoming more widespread despite the reservations of some (13), and the optimal method of perineal wound closure remains a topic of discussion. The reported results of primary closure of the perineal defect are poor (34) and most surgeons performing ELAPE opt for an adjunct (35). The literature analyzed suggests that perineal closure using a biologic mesh produces wound infection and complication rates that are comparable to other methods of reconstruction, such as myocutaneous flaps. Myocutaneous flap reconstruction using a vertical rectus abdominis (VRAM), gracilis, or the gluteus maximus, however, has short-term disadvantages, such as longer operative times and the need for plastic surgical expertise, resulting in higher operative costs, flap necrosis, wound complications at the donor site, and longer bed rest (15). Longer term incisional hernias at the VRAM donor site and reduced abdominal wall strength have been reported (36). Biologic mesh reconstruction avoids all of these potential complications.

Synthetic non-absorbable mesh is associated with high infection rate in contaminated fields and consequently is considered by many to be contra-indicated for use in perineal reconstruction following ELAPE (37). The role of newer, absorbable synthetic meshes is, as yet, unclear. Biologic meshes are composed of an acellular collagen matrix that is believed to allow tissue regeneration, neovascularization, repopulation with fibroblasts, and therefore provides a scaffold for tissue incorporation (15, 23). This is thought to reduce the rate of infection. However, the overall volume and quality of evidence available regarding biologic mesh use for perineal reconstruction following ELAPE is poor, with observational retrospective studies predominating. There have been some attempts at comparative studies, but these too have been of low quality with a high risk of bias and confounding factors. Head-to-head randomized trials or high-quality prospective cohort studies comparing biological with synthetic mesh, types of biologic mesh, and biologic mesh with (myo)fasciocutaneous flaps are also lacking, partly because there is no consensus among surgeons as to the optimal biologic mesh or optimal tissue flap. Trials directly comparing any technical adjunct to primary closure alone as a control arm may be difficult to perform in light of the lack of equipoise among surgeons and possibly even unethical given the reported poor results of primary closure. Furthermore, there does not appear to be a consensus in the studies regarding perineal outcome reporting. There are a variety of different end points recorded across the studies, such as perineal defect size, blood loss, and operating time. There needs to be a focus on standardized definitions and reporting of perineal healing rates, perineal hernia, and functional outcomes following ELAPE (38).

Jensen et al. also examined the long-term follow-up for patients undergoing pelvic floor reconstruction with a biologic mesh following ELAPE (25). As well as low perineal hernia rates, there was no major restriction in movement or sitting. Chronic pain had resolved in all patients at a median of 8 months, and there was no major limitation to walking. However, other studies evaluating quality-of-life scores using validated tools (11) demonstrated a favorable comparison to the reference population of patients with colorectal cancer who had undergone a standard APE, whereas patients who had undergone flap reconstruction had a lower quality of life score (11).

Of note, a number of studies from Beijing have been included for analysis. The three studies include patients managed over an approximately 3-year period, and there is overlap of the studies within the time period, therefore suggesting some replication. One study is classified as a case series (23), the second a RCT (14), and the third another case series (24). It is unclear as to whether these three studies are from the same patient group or three different cohorts.

## Conclusion

Overall, the use of a biologic mesh to close perineal defects has comparable complications rates to myocutaenous flaps but may offer advantages, such as shorter operating time and early mobilization, which results in a more cost-effective repair (15). Biologic mesh-assisted perineal reconstruction is a promising technique to improve wound healing, but there is not enough evidence to support its use in all patients who have undergone ELAPE. The results from high-quality prospective RCTs or national/international collaborative audits using statistical process control as a methodology of assessment of improvement are required.

## Author Contributions

All authors listed have made substantial, direct, and intellectual contribution to the work and approved it for publication.

## Conflict of Interest Statement

The authors declare that the research was conducted in the absence of any commercial or financial relationships that could be construed as a potential conflict of interest.
